# Optimized Test Utilization Significantly Increased the Positive Detection Rate for Myeloproliferative Neoplasms

**DOI:** 10.3390/cancers18142282

**Published:** 2026-07-16

**Authors:** Landry E. Nfonsam, Melody (Zhenchen) Qi, Darci T. Butcher, Clinton J. V. Campbell, Amanda Cocca, Shreyash Dalmia, Gwynivere A. Davies, Betty-Ann Hohenadel, Elizabeth McCready

**Affiliations:** 1 Hamilton Regional Laboratory Medicine Program, Hamilton Health Sciences, Hamilton, ON L8V 1C3, Canada; 2 Department of Pathology and Molecular Medicine, McMaster University, Hamilton, ON L8S 4K1, Canada; 3 Biology and Pharmacology Co-Op Program, McMaster University, Hamilton, ON L8S 4L8, Canada; 4 Department of Oncology, McMaster University, Hamilton, ON L8S 4L8, Canada; 5 Juravinski Cancer Center, Hamilton Health Sciences, Hamilton, ON L8V 5C2, Canada; 6 Escarpment Cancer Research Institute, Hamilton, ON L8V 5C2, Canada

**Keywords:** myeloproliferative neoplasm, test utilization, diagnostic testing, *BCR::ABL1*, *JAK2*, *CALR*, *MPL*

## Abstract

Background: Molecular testing for myeloproliferative neoplasms (MPNs) without defined clinical criteria can lead to inefficient laboratory utilization without proportional diagnostic benefit. Methods: We evaluated the impact of implementing clinical acceptance criteria, including unexplained abnormal blood counts, unusual-site thrombosis, unexplained hepatosplenomegaly, and/or leukoerythroblastic blood film for gene (*JAK2*, *CALR*, *MPL* and/or *BCR::ABL1*) testing of patients with suspected MPNs in Hamilton, Canada and surrounding areas. The impact of testing criteria on ordering practices and diagnostic yields was assessed by retrospective examination of 3590 MPNs test requests submitted between 1 January and 31 December 2025; including 5.5 months of permissive testing (pre-implementation period), 2 months with a transition in testing criteria (grace period) and 4.5 months with restricted testing (post-implementation period). During the post-implementation period, the Laboratory also transitioned from sequential single-gene PCR-based assays to an MPN next-generation sequencing (NGS) panel for DNA-based MPN testing. Results: Our results show that test volumes decreased significantly from 1835 in the pre-implementation period to 1050 post-implementation, while the positivity rate increased from 12% to 17% (χ^2^ = 12.26, *p* = 0.001). Patients with elevated platelet counts (237 of 1050 tested) demonstrated the highest positivity rate of 35% (*n* = 83/237). *JAK2* V617F represented the highest proportion of all detected mutations at 73% (*n* = 127/174). Conclusions: These findings show that implementing appropriate pre-test clinical criteria significantly improved MPN test utilization and diagnostic yield.

## Simple Summary

Although molecular testing for myeloproliferative neoplasms (MPNs)-associated genetic variants can facilitate diagnosis by establishing the presence of an abnormal clonal cell population, inappropriate utilization of these tests can lead to unsustainable laboratory workloads, decreased test efficiency and lower variant detection frequencies. This manuscript provides a unique assessment of the impact of clinical testing indications on MPN molecular testing by comparing test volumes and detection rates before and after implementation of evidence-based testing criteria. The testing criteria were established to prioritize testing of patients most likely to have MPNs. Strict enforcement of criteria, combined with an MPN driver NGS-based testing strategy, resulted in significant improvements to the efficiency and diagnostic yield of molecular testing. These findings highlight the importance of quality improvement and test utilization assessment in diagnostic laboratory testing and supports a scalable model in which clinical gatekeeping precedes comprehensive molecular evaluation, offering a sustainable framework for precision diagnostics within healthcare.

## 1. Introduction

Recent updates to the World Health Organization (WHO) and International Consensus Classification (ICC) frameworks for myeloid and lymphoid neoplasms highlight molecular testing as an integral tool in the diagnosis and classification of myeloid neoplasms. This is particularly true for *BCR::ABL1*-negative myeloproliferative neoplasms (MPNs) including polycythemia vera (PV), essential thrombocythemia (ET), and primary myelofibrosis (PMF) [1,2,3]. These entities are typically driven by somatic mutations in genes affecting the JAK-STAT signaling pathway, including *JAK2*, *CALR*, or *MPL* [1,4,5]. Although these canonical driver mutations constitutively activate JAK-STAT signaling and promote myeloid proliferation, they may be insufficient alone to initiate or sustain disease [6,7]. Studies suggest that these signaling mutations may cooperate with recurrent mutations in epigenetic regulators, including TET2, ASXL1, EZH2, DNMT3A, and IDH1/2, to influence stem cell self-renewal, clonal evolution, disease phenotype, and leukemic transformation [8,9,10,11,12]. These findings highlight the complex molecular landscape of MPNs and underscore the need for integrated genomic approaches to improve diagnosis, prognosis, and therapeutic stratification, while distinguishing clonal from reactive, secondary, or inherited causes of blood count abnormalities [1,6].

In response to consensus provincial recommendations for MPN diagnostic testing in Ontario, Canada [13], our laboratory previously adopted a broad testing approach in which test requests for suspected MPN or chronic myeloid leukemia (CML) prompted concurrent DNA-based driver mutation testing (*JAK2*, *CALR* and *MPL*) and RNA-based *BCR::ABL1* fusion transcript testing through a bundled ordering configuration, independent of specific clinical discriminators. DNA testing was performed sequentially via single analyte tests (*JAK2* p.V617F then *CALR* in bone marrow and peripheral blood samples, followed by *MPL* and *JAK2* exon 12 testing of bone marrow samples only) with discontinuation of downstream testing if a driver mutation was detected. Given overlapping phenotypic spectrums of MPNs and CML, this bundled ordering configuration was designed to facilitate more sensitive diagnosis through the detection of clonal genetic variants in patients that may not otherwise be tested by more restrictive test algorithms. However, this approach can also lead to unnecessary testing, inflated test volumes, elevated frequency of negative results, inefficient use of laboratory resources, and potential delayed results for patients with a higher likelihood of clonal disease. Moreover, while sequential DNA testing reduces redundant testing when a driver mutation has already been identified, it can also limit the detection of rare variants occurring in the genes not tested. In a publicly funded healthcare system like Canada’s, these inefficiencies pose sustainability challenges. Laboratory diagnostic stewardship strategies have the potential of improving test utilization through the implementation of clinical acceptance criteria and/or assay consolidation through multi-gene testing platforms such as next-generation sequencing (NGS).

In June 2025, the Hamilton Health Sciences (HHS) Clinical Genetics Laboratory, in collaboration with hematology and hematopathology partners, defined and implemented clinical criteria for testing that would facilitate streamlined identification of patient populations with a high pre-test probability of MPNs or CML and most likely to benefit from available molecular genetic testing. To further improve testing efficiency and for diagnostic completeness, including capture of non-canonical driver mutations for MPNs, in August 2025, the HHS laboratory also transitioned from sequential single-gene PCR-based assays to an NGS-first workflow which consolidated multiple MPN targets into a single gene panel for rapid testing of 12 targets (*JAK2*, *CALR*, *MPL*, *SF3B1*, *CSF3R*, *KIT*, *TP53*, *IDH1*, *IDH2*, *CEBPA*, *NPM1* and *FLT3*). This report evaluates the impact of these interventions on MPN laboratory test utilization and diagnostic yield. The effectiveness of this continuous quality improvement project was evaluated by measuring the impact of these interventions on MPN laboratory test utilization and diagnostic yield.

## 2. Materials and Methods

### 2.1. Study Design and Data Source

Molecular test data that include specimen ID, specimen date of receipt, sample indication (reason for referral) and test result from 1 January to 31 December 2025 were extracted from the laboratory information system at HHS and retrospectively analyzed. Patient date-of-birth and specimen type were also extracted, but not analyzed except to facilitate linkage of different concurrent test requests from the same individual. Only diagnostic testing for suspected MPNs was considered in the study. Data from comprehensive gene panel testing (including testing of 39 myeloid neoplasia-associated genes) for confirmed MPNs were excluded from the current study. Extracted data were divided into 3 categories based on the time period when new mandatory clinical acceptance criteria for testing were implemented. These time periods include:

- Pre-implementation period (1 January to 15 June 2025): This represents the period before new clinical acceptance criteria for MPN testing were implemented (i.e., baseline). During this period, no specific clinical acceptance criteria were required, and reflex/combined DNA-RNA-based testing was permitted. DNA testing (peripheral blood or bone marrow) entailed a sequential approach [14], beginning with *JAK2*:c.1849G>T, p.(Val617Phe) (*JAK2* V617F) by allele specific polymerase chain reaction (PCR), reflexing to *CALR* exon 9 by PCR and fragment analysis and/or Sanger sequencing if *JAK2* V617F was negative, and then to *MPL* or *JAK2* exon 12 by Sanger sequencing if requested (from bone marrow only). RNA was used for *BCR::ABL1* fusion transcript testing by reverse transcription (RT)-PCR. Both DNA- and RNA-based tests were performed for all suspected MPN investigations.
- Grace period (16 June to 11 August 2025): This represents the period immediately after new clinical acceptance criteria were implemented. During this period, suspected *BCR::ABL1*-positive MPN RNA-based testing was decoupled from suspected *BCR::ABL1*-negative MPN DNA-based workup, and restricted only to cases meeting specific *BCR::ABL1* testing criteria. For requests requiring DNA-based *JAK2*, *CALR* or *MPL* testing (suspected *BCR::ABL1*-negative MPN), provision of at least one of the following criteria was requested to facilitate test initiation; unexplained abnormal blood counts (leukocytes ≥ 11 × 10^9^/L, hemoglobin ≥ 160 g/L and/or platelets ≥ 400 × 10^9^/L), unexplained unusual-site thrombosis or unexplained hepatosplenomegaly and/or leukoerythroblastic blood film. For RNA-based *BCR::ABL1* orders (suspected *BCR::ABL1*-positive MPN), unless the patient had a confirmed chronic myeloid leukemia (CML) diagnosis, testing was encouraged for patients with a complete blood count (CBC) showing leukocyte counts ≥ 11 × 10^9^/L with left shift (elevated leukocytes with left shift) or platelet counts ≥ 400 × 10^9^/L with basophilia (elevated platelets with basophilia). For either DNA-based or RNA-based testing requests, multiple indications could be provided. Multiple indications could also be provided that prompt both DNA-based and RNA-based testing for cases where it is unclear whether the patient is suspected of being *BCR::ABL1*-positive or *BCR::ABL1*-negative MPN. Providing these clinical indications at the time of test ordering was encouraged, although it was not required during the grace period. Requests meeting the “BCR::ABL1” (elevated leukocytes with left shift or elevated platelets with basophilia) criteria, along with those that did not meet the above recommendations, were grouped into an “All Others” category. This category also included cases in which criteria were not provided by the ordering physician, criteria were not selected on the requisition, outdated requisition forms were used, special consideration was granted following patient chart review, or testing was approved at the discretion of the laboratory scientist.
- Post-implementation period (12 August to 31 December 2025): This represents the period after which the provision of required acceptance criteria was enforced with systematic rejection (test suspended or discontinued) of non-indicated requests using the above criteria. Requests not meeting criteria were reported as “Testing suspended,” accompanied by a clear statement that testing would be reactivated if relevant criteria were provided. All test requests received were manually reviewed to ensure that an appropriate indication was selected on the requisition by the ordering physician according to the same criteria described for the grace period. Samples without relevant indications were stored and testing could be reactivated by the referring physician, if clinically indicated, and in accordance with the outlined criteria. During the post-implementation period, the Laboratory also transitioned its DNA-based MPN test from the sequential single-gene PCR-based approach to an NGS-first workflow using an MPN Core NGS panel that includes targeted testing of *JAK2*, *CALR*, *MPL*, *SF3B1*, *CSF3R*, *KIT*, *TP53*, *IDH1*, *IDH2*, *CEBPA*, *NPM1* and *FLT3* (Oxford Gene Technology Ltd., United Kingdom). The MPN Core NGS panel enabled the concurrent detection of canonical and clinically actionable drivers, as well as additional relevant non-canonical variants.

### 2.2. Data Analyses and Statistics

We assessed laboratory test utilization in the pre-implementation, grace and post-implementation periods, by evaluating all DNA-only, RNA-only, DNA-RNA and ‘No indication’ requests that were sent to the genetics laboratory, for positivity rates (diagnostic yield), as well as rejection rates. A sample was considered positive, and reported only if a Tier I variant (pathogenic or likely pathogenic) was detected [15]. Variants of uncertain clinical significance were not reported. Positivity rates were compared across the pre-implementation, grace and post-implementation periods using chi-square (χ^2^) testing. Analyses were performed using SciPy library (v.1.12.0); two-sided *p* < 0.05 was considered statistically significant.

## 3. Results

### 3.1. DNA-Only Testing Increased Significantly Post New Policy Implementation

A total of 3590 molecular tests were requested between 1 January and 31 December 2025 (Table 1). In the pre-implementation period, 1910 tests were requested, of which 1835 (96.1%) were completed and 75 (3.9%) were discontinued, primarily due to duplicate or previously completed requests. During the grace period, 455 tests were requested, with 427 (93.8%) completed and 28 (6.2%) discontinued, again largely reflecting duplication or prior completion. In the post-implementation period, 1225 tests were requested, of which 1050 (85.7%) were completed and 175 (14.3%) discontinued. In contrast to earlier periods, discontinued requests in the post-implementation period were predominantly due to missing indications.

The ordering pattern was markedly different between the pre-implementation, grace and post-implementation periods. Of the 1910 tests that were requested in the pre-implementation period, the largest proportion was concurrent DNA-RNA (*n* = 781/1910: 40.9%), followed by DNA-only (*n* = 550/1910: 28.8%) and then RNA-only (*n* = 504/1910: 26.4%) (Table 2). Only 3.9% (*n* = 75/1910) of test requests resulted in test rejection during this period. During the grace period, concurrent DNA-RNA test requests decreased to 18.5% (*n* = 84/455), with DNA-only testing becoming more common (*n* = 252/455: 55.4%). This pattern persisted post-implementation policy, with concurrent DNA-RNA testing further reducing to 6.7% (*n* = 82/1225), RNA-only test requests decreasing to 16.9% (*n* = 207/1225), and DNA-only testing predominating at 62.1% (*n* = 761/1225). The number of test rejections increased in the post-implementation period to 14.3% (*n* = 175/1225), reflecting systematic rejection of non-indicated requests.

### 3.2. Positive Test Results Increased Significantly in the Post-Implementation Period

**Figure 1:**
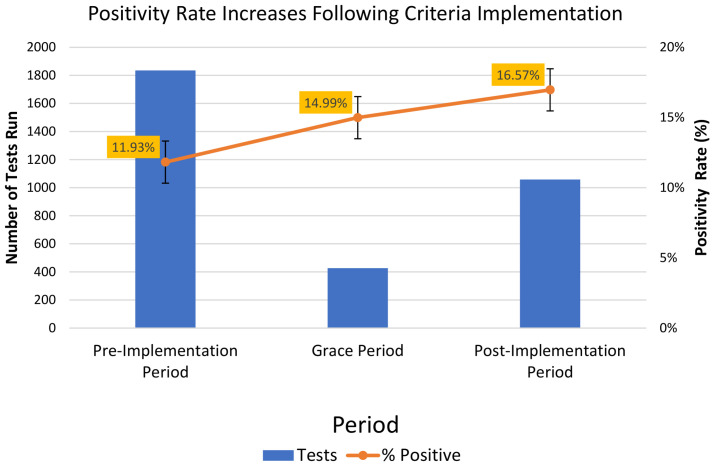
Positivity rate increased significantly in the post-implementation period, despite decreased test volume. Bar chart showing the total number of molecular tests performed during the pre-implementation, grace, and post-implementation periods, with an overlaid line (orange) indicating the positivity rate for each policy period. Percent positive rates are labeled directly on the figure. Despite a reduction in overall test volumes following implementation of mandatory clinical criteria, diagnostic yield increased from 11.93% in the pre-implementation period to 16.57% in the Post-implementation Period. Comparison of the pre-implementation to the post-implementation period demonstrated a statistically significant increase in positivity rate (χ^2^ = 12.3, *p* = 0.001).

To evaluate the impact of implementing the new mandatory clinical acceptance criteria for suspected MPN testing on laboratory efficiency, the overall positivity rate (diagnostic yield) was compared against test volumes across the three implementation periods. Despite a marked reduction in overall test volume, the positivity rate increased progressively across the grace and post-implementation periods (Table 3, Figure 1). The pre-implementation period demonstrated a baseline positivity rate of 11.9% (*n* = 219/1835). This increased to 15.0% (*n* = 64/427) during the grace period, and to 16.6% (*n* = 174/1050) in the post-implementation period. The increase from pre- to post-implementation period was statistically significant (χ^2^ = 12.3, *p* = 0.001). The overall increase in positivity rate following implementation was primarily driven by higher detection rates for *JAK2* V617F, *MPL*, and *BCR::ABL1*, which increased from 9.0% (166/1835) to 12.1% (127/1050), 0.2% (3/1835) to 0.6% (6/1050), and 1.5% (27/1835) to 2.5% (26/1050), respectively. In contrast, positivity rates for *JAK2* exon 12 and *CALR* remained largely unchanged, with *JAK2* exon 12 increasing from 0.1% (2/1835) to 0.2% (2/1050) and *CALR* from 1.1% (21/1835) to 1.2% (13/1050).

### 3.3. Elevated Platelet Count Revealed the Highest Positivity Rate

To identify specific drivers of test utilization and diagnostic efficiency, testing during the grace and post-implementation periods was stratified by the provided clinical criteria. During the grace period, “Elevated Platelets” demonstrated the highest yield with 37.9% (*n* = 22/58) positive mutations, which included 16 *JAK2* V617F and 6 *CALR* positives (Table 3). The lowest yields were observed for “Elevated Leukocytes” and “Unusual-Site Thrombosis” at 6.7% (*n* = 1/15) and 3.6% (*n* = 1/28), respectively. All other indications provided by the ordering physicians, including “BCR::ABL1” (elevated leukocytes with left shift or elevated platelets with basophilia), were combined into the “All Other” category and accounted for 12.2% (*n* = 34/278) positivity rate.

In the post-implementation period, “Elevated Platelets” remained the highest-yield criteria with 35.0% (*n* = 83/237) positives and demonstrated a broad mutation spectrum (68 *JAK2* V617F, 9 *CALR*, 4 *MPL*, and 2 *BCR::ABL1* (both DNA and RNA tested) (Table 3). This was followed by “Unexplained Hepatosplenomegaly and/or Leukoerythroblastic Blood Film”, “Elevated Leukocytes”, “Elevated Hemoglobin” and then “Unusual-Site Thrombosis” at 26.1% (*n* = 6/23), 17.9% (*n* = 15/84), 7.4% (20/272) and 7.1% (*n* = 9/126), respectively. As expected, *BCR::ABL1* fusion transcripts were the most frequently detected variant in the RNA-based “BCR::ABL1 (Elevated leukocytes with left shift or elevated platelets with basophilia)” category, constituting 17 of the 20 positives (85.0%), out of 214 tested (9.3%, *n* = 20/214). Three positives (15.0%) in this category were *JAK2* V617F (both DNA and RNA tested). In addition, two *JAK2* exon 12 variants were detected in post-implementation without reliance on sequential reflex pathways.

## 4. Discussion

This study represents a comprehensive quality improvement initiative aimed at optimizing molecular diagnostic testing for patients in Hamilton and the surrounding areas with suspected MPNs. Optimizing testing for MPNs is essential in reducing inefficient laboratory utilization while maximizing the proportional diagnostic benefit [16,17]. This retrospective evaluation demonstrates that implementing evidence-based clinical acceptance criteria for MPN testing significantly improves molecular diagnostic yield by reducing low-value testing and increasing positivity rate. The key operational benefit was the elimination of concurrent DNA-RNA testing for all suspected MPN test requests and the rejection of requests lacking clinical justification.

The significant increase in diagnostic yield (11.9% to 16.6%) following new test criteria implementation, without a significant change in the overall number of MPN-associated variant detected (219 variants in 166 days in the pre-analytical period versus (174 variants in 142 days in the post analytical period), suggested that the new process disproportionately removed test requests that are less likely to be clonal (as defined by the detection of a clonal MPN associated genetic variant). In hematology, reactive leukocytosis or thrombocytosis is common, and distinguishing reactive from clonal etiologies is a frequent diagnostic challenge [18]. By introducing objective acceptance thresholds and requiring explicit indications, the laboratory reduced unnecessary test requests, thus preserving capacity for higher-yield testing. Additionally, although no formal economic analysis was conducted, implementation of pre-test criteria led to a 43% reduction in testing volume (from 1835 to 1050), substantially decreasing molecular assay use, technologist workload, reagent consumption, and interpretation time. In a publicly funded healthcare system, this reduction in low-yield testing likely translates into cost savings and improved laboratory efficiency and capacity.

Detection of the highest diagnostic yield in patients with elevated platelet counts correlates, in part, with known biology and genetics of the disease [19,20]. ET, prefibrotic PMF and PV often present with thrombocytosis (elevated platelet count), and driver mutations in *JAK2*, *CALR* and *MPL* are common in these clonal diseases [21,22,23,24]. Although the reported prevalence of *JAK2* V617F mutation in patients with ET (elevated platelets) approaches 50–60%, the lower yield of 35–38% observed in our unselected cohort likely reflects the high prevalence of reactive thrombocytosis in routine clinical practice [21,25,26]. Nonetheless, these data confirm that unexplained thrombocytosis remains an important prompt for molecular evaluation of MPNs [21,27,28].

A small subset of post-implementation period test requests for both “suspected *BCR::ABL1*-negative MPN” and “suspected *BCR::ABL1*-positive MPN (elevated leukocytes with left shift or elevated platelets with basophilia)” were *JAK2* V617F positive (*n* = 3/214: 1.4%). These findings are best interpreted as a diagnostic challenge in terms of pre-test probability rather than policy failure, and reflect the well-recognized clinical overlap between *BCR::ABL1*-positive and *BCR::ABL1*-negative MPNs. None of these cases demonstrated concurrent *JAK2*-*BCR::ABL1* driver mutations, consistent with their expected typical mutual exclusivity [29,30,31], although cases with dual *BCR::ABL1* and JAK2 mutation have been described as rare events in the literature [29,30,31,32]. Identification of *JAK2* mutations in these patients is highly impactful to diagnosis and clinical management given the distinct therapeutic approaches required for *BCR::ABL1*-positive versus *BCR::ABL1*-negative MPNs.

The grace period served as an important transition period for ordering providers to learn about the new MPN test request process. As expected, only partial test improvement was observed during this period, aligning with the idea that strict enforcement of new ordering policies should have a durable, consistent change [33]. Accordingly, adherence by clinicians increased in the post-implementation period following stricter pre-test criteria implementation.

Beyond utilization management, the transition to an NGS-first workflow provided an important clinical and operational advantage by consolidating multiple MPN targets into a single assay, reducing reliance on sequential reflex testing and minimizing the risk of incomplete diagnostic evaluation. However, because implementation of the NGS-first workflow coincided with the introduction of mandatory clinical acceptance criteria, the individual contribution of each intervention cannot be completely separated. Nevertheless, the NGS assay demonstrated 100% concordance with the previous sequential reflex approach during clinical validation and therefore primarily streamlined the testing process rather than increasing analytical sensitivity. The NGS gene panel used includes nine genes not previously tested in the sequential PCR-based test (*SF3B1*, *CSF3R*, *KIT*, *TP53*, *IDH1*, *IDH2*, *CEBPA*, *NPM1* and *FLT3*). These extra genes were included in the panel for concurrent rapid testing of relevant variants in patients with a new diagnosis of AML or high-grade MDS. There were no variants detected in *SF3B1*, *CSF3R*, *KIT*, *TP53*, *IDH1*, *IDH2*, *CEBPA*, *NPM1* nor *FLT3* in patients with suspected MPNs from the current cohort. Furthermore, only two non-canonical *JAK2* variants were identified in the post-implementation period, contributing negligibly to the overall positivity rate. These findings suggest that the observed improvement in diagnostic yield was driven predominantly by enhanced patient selection through clinical gatekeeping, while the principal benefit of NGS was improved laboratory efficiency and positioning the HHS Genetics Laboratory to support future molecular risk stratification frameworks. Future studies may include evaluating the combinatorial effects of different clinical acceptance criteria on the positivity rate and clinical outcomes to further refine and validate the proposed testing algorithm. Data on clinically significant MPN diagnoses outside of the acceptance criteria were not collected for this study and, therefore, were not evaluated. Future studies may also include a formal evaluation of implementing pre-test criteria on cost savings, investigating the impact of key demographic variables, such as age, sex, and ethnicity, on diagnostic yield, as well as evaluating a longer time period (beyond 4.5 months post-implementation) and a larger dataset to determine whether these factors can further optimize patient selection for molecular testing. It is also important to note that this study was conducted within a single regional healthcare system in Hamilton, Canada; therefore, the implemented criteria may have limited applicability to other healthcare settings with different patient populations and testing practices. As such, a broader adoption by other clinical laboratories should be approached with caution. However, we hope our findings will encourage further discussion among Canadian laboratories offering MPN testing regarding the potential implementation of similar criteria.

## 5. Conclusions

In summary, the enforcement of evidence-based testing criteria combined with an MPN driver NGS-based testing strategy significantly improved the efficiency and diagnostic yield of molecular testing for MPNs. These findings support a scalable testing model in which clinical gatekeeping precedes comprehensive molecular evaluation, offering a sustainable framework for precision diagnostics within healthcare.

## Figures and Tables

**Table 1:** Test Volumes during the pre-implementation, grace, and post-implementation periods.

Indication Group		Period (Number of Orders)		
	Pre-Implementation Period (*n* = 1910)	Grace Period (*n* = 455)	Post-Implementation Period (*n* = 1225)	Total: 3590
Elevated Platelets	Not Applicable	58	237	
Elevated Hemoglobin	Not Applicable	43	272	
Elevated Leukocytes	Not Applicable	15	84	
Unusual-Site Thrombosis	Not Applicable	28	126	
HepSplen/leukoerythro	Not Applicable	5	23	
Special Consideration	Not Applicable		94	
BCR::ABL1 (ELS or EPB)	Not Applicable		214	
All others	Not Applicable	278		
Total Tested	1835 (96%)	427 (94%)	1050 (86%)	
Test Suspended: Previously tested/duplicate	75	28	56	
Policy Rejection: Insufficient Indication			119	

Abbreviations: HepSplen/leukoerythro: Unexplained Hepatosplenomegaly and/or leucoerythroblastic blood film; ELS or EPB: Elevated leukocytes with left shift or elevated platelets with basophilia; All Others: Indicated criteria not provided by the ordering physician, criteria not selected on the requisition, outdated requisition forms used, special consideration following patient chart review, or testing approved at the discretion of the laboratory scientist.

**Table 2:** DNA-only requests increased significantly post-criteria implementation.

Test Type	Period (Number of Orders)		
	Pre-Implementation Period (*n* = 1910)	Grace Period (*n* = 455)	Post-Implementation Period (*n* = 1225)
DNA-Only	550 (28.8%)	252 (55.4%)	761 (62.1%)
RNA-Only	504 (26.4%)	91 (20%)	207 (16.9%)
DNA and RNA Performed	781 (40.9%)	84 (18.5%)	82 (6.7%)
No Molecular Testing Performed	75 (3.9%)	28 (6.2%)	175 (14.3%)

**Table 3:** Positivity rates and volumes during the pre-implementation, grace, and post-implementation periods based on the indicated criteria. Positive results increased significantly post-criteria implementation.

**Pre-Implementation Period (*n* = 1910)**								
**Indication Group**	**JAK2 V617F+**	**JAK2 Exon 12+**	**CALR+**	**MPL+**	**BCR::ABL1+**	**Negatives**	**Total Positives**	**Yield %**
Not Applicable								
Total Tested (1835)	166	2	21	3	27	1616	219	11.93%
**Grace Period (*n* = 455)**								
**Indication Group**	**JAK2 V617F+**	**JAK2 Exon 12+**	**CALR+**	**MPL+**	**BCR::ABL1+**	**Negatives**	**Total Positives**	**Yield %**
Elevated Platelets (58)	16	0	6	0	0	36	22	37.93%
Elevated Hemoglobin (43)	5	0	0	0	0	38	5	11.63%
Elevated Leukocytes (15)	1	0	0	0	0	14	1	6.67%
Unusual-Site Thrombosis (28)	1	0	0	0	0	27	1	3.57%
HepSplen/leukoerythro (5)	0	0	1	0	0	4	1	20%
All Others (278)	24	0	3	0	7	244	34	12.23%
Total Tested (427)	47	0	10	0	7	363	64	14.99%
**Post-Implementation Period (*n* = 1230)**								
**Indication Group (Number Tested)**	**JAK2 V617F+**	**JAK2 Exon 12+**	**CALR+**	**MPL+**	**BCR::ABL1+**	**Negatives**	**Total Positives**	**Yield %**
Elevated Platelets (237)	68	0	9	4	2	154	83	35.0%
Elevated Hemoglobin (272)	20	0	0	0	0	252	20	7.35%
Elevated Leukocytes (84)	7	0	1	1	6	69	15	17.86%
Unusual-Site Thrombosis (126)	9	0	0	0	0	117	9	7.1%
HepSplen/leukoerythro (23)	5	0	1	0	0	17	6	26.09%
Special Consideration (94)	15	2	2	1	1	73	21	22.3%
BCR::ABL1 (ELS or EPB) (214)	3	0	0	0	17	194	20	9.35%
Total Tested (1050)	127	2	13	6	26	876	174	16.57%

Abbreviations: HepSplen/leukoerythro: Unexplained Hepatosplenomegaly and/or leukoerythroblastic blood film; ELS or EPB: Elevated leukocytes with left shift or elevated platelet with basophilia; All Others: Indicated criteria not provided by the ordering physician, criteria not selected on the requisition, outdated requisition forms used, special consideration (borderline indications) following patient chart review and/or consultation with the hematologist/hematopathologist, or testing approved at the discretion of the laboratory scientist.

## Data Availability

Available from the corresponding author upon reasonable request.
